# Connexins and Pannexins in Vascular Function and Disease

**DOI:** 10.3390/ijms19061663

**Published:** 2018-06-05

**Authors:** Filippo Molica, Xavier F. Figueroa, Brenda R. Kwak, Brant E. Isakson, Jonathan M. Gibbins

**Affiliations:** 1 Department of Pathology and Immunology, University of Geneva, CH-1211 Geneva, Switzerland; filippo.Molica@unige.ch; 2 Departamento de Fisiología, Faculdad de Ciencias Biológicas, Pontifica Universidad Católica de Chile, Santiago 8330025, Chile; xfigueroa@bio.puc.cl; 3 Robert M. Berne Cardiovascular Research Center, University of Virginia School of Medicine, Charlottesville, VA 22908, USA; bei6n@virginia.edu; 4 Department of Molecular Physiology and Biophysics, University of Virginia School of Medicine, Charlottesville, VA 22908, USA; 5 Institute for Cardiovascular & Metabolic Research, School of Biological Sciences, Harborne Building, University of Reading, Reading RG6 6AS, UK; j.m.gibbins@reading.ac.uk

**Keywords:** connexin, pannexin, vascular physiology, vascular disease

## Abstract

Connexins (Cxs) and pannexins (Panxs) are ubiquitous membrane channel forming proteins that are critically involved in many aspects of vascular physiology and pathology. The permeation of ions and small metabolites through Panx channels, Cx hemichannels and gap junction channels confers a crucial role to these proteins in intercellular communication and in maintaining tissue homeostasis. This review provides an overview of current knowledge with respect to the pathophysiological role of these channels in large arteries, the microcirculation, veins, the lymphatic system and platelet function. The essential nature of these membrane proteins in vascular homeostasis is further emphasized by the pathologies that are linked to mutations and polymorphisms in Cx and Panx genes.

## 1. Introduction

The cardiovascular system consists of the heart pumping the blood to a closed circuit of interconnected blood vessels, allowing for the indispensable and constant supply of O_2_ and vital nutriments to every single tissue throughout the human body. The blood contains components, such as coagulation factors and platelets, that are essential to keeping the cardiovascular circuit closed after injury by initiating haemostasis and formation of a platelet clot. The systemic circulation is composed of large elastic arteries such as the aorta, serving as high-pressure conduits for the blood to smaller muscular arteries and arterioles. Arterioles are resistance arteries controlling blood flow into capillary beds by their high vasodilatory and vasoconstrictive ability. In the capillary beds, the actual exchange of O_2_/CO_2_, nutriments, catabolites and fluid takes place between the blood and the surrounding tissue. The blood returns to the heart via venules and veins. The excess of interstitial fluid returns to the systemic circulation via lymphatics, a blind-ended system of lymphatic capillaries converging into collecting vessels and ending into the subclavian vein. Much evidence has demonstrated an important role for connexins (Cxs) and pannexins (Panxs) in many aspects of vascular physiology and pathology. In this review, we will focus on the role of Cxs and Panxs in the physiology/pathophysiology of the vascular system by systematically following the route of the blood from the left ventricle through the systemic circulation to its way back to heart.

## 2. Connexins and Pannexins

Cxs belong to a family of 20 to 21 proteins expressed in a wide variety of tissues [1]. Cx genes are separated into 5 subfamilies according to their sequence homologies [2]; most cardiovascular Cxs are found in the α subfamily (for instance, *GJA4*). The names of Cx proteins, on the other hand, are determined by their specific molecular weight in kDa (for instance, Cx37). Structurally, Cxs comprise 4 α-helical transmembrane domains (TM1–TM4) and two extracellular loops (EL1 and EL2) that are highly conserved among the family members. Substantial differences among Cxs, both in length and composition, are found in their cytoplasmic amino-terminal (NT) and carboxy-terminal (CT) parts, as well as in the intracellular loop (IL). The synthesis of Cxs occurs in the endoplasmic reticulum (ER) and their oligomerization in the ER/Golgi or trans-Golgi network results in the formation of hexameric connexons [3,4]. Then, connexons traffic to the plasma membrane along microtubules. When the membranes of two cells are in close proximity, connexons from one cell can connect with their corresponding parts in the adjacent cell and form gap junction channels, which permit the intercellular exchange of ions and metabolites up to ~1kDa. Connexons are normally closed but may operate in a pathological setting as hemi-channels enabling the transmembrane passage of Ca^2+^, ATP and glutamate for instance [5,6]. Cx channel gating is critically regulated by a number of factors, including voltage, pH and Ca^2+^ and post-translational modifications such as phosphorylation [7,8]. The “connexin interactome”, a protein interacting network with the Cx as central mediator [9,10], has been receiving increasing attention in recent years. For example, an interacting complex of gap junctions, desmosomes and Na^+^ channels that cooperate to control excitability, electrical coupling and intercellular adhesion are found at intercalated discs in the heart [10,11]. The plethora of diseases associated with mutations and polymorphisms in Cx genes further underlines the crucial role of these structures in tissue homeostasis [12,13].

Pannexins (Panxs) represent a smaller family of 3 transmembrane proteins (Panx1–3) exhibiting a topology similar to Cxs but no sequence homology [14,15,16]. The glycosylation of specific sites in the ELs of Panx1 and Panx3 likely prevents docking of pannexons [16,17], and it is presumed that pannexons act as single-membrane channels connecting the cytoplasm to the extracellular compartment. While Panx2 and Panx3 display a rather limited expression pattern (central nervous system for Panx2 and bones and skin for Panx3), Panx1 shows a ubiquitous expression pattern and is thus also found in vascular cells. Similar to Cx hemi-channels, pannexons serve as “communication channels” by permitting the release of small molecules, for instance purines, that subsequently signal via the activation of membrane receptors in neighboring cells or even at distance. An important difference between Cx hemi-channels and Panx channels is that the latter can be opened at physiological membrane potential and physiological intra- and extra-cellular Ca^2+^ concentration by, for example, mechanical stretching or upon activation of purinergic P2 receptors [18]. Instead, Cx hemi-channels only become functional under conditions associated with pathologies such as hypoxia or ischemia and will be only briefly mentioned in this review. Excellent reviews on Cx hemi-channels have been published recently [19,20,21].

## 3. Role of Cxs and Panxs in Distributing Arteries and Atherosclerosis

The largest distributing arteries are elastic vessels, which allows them to receive a high and pulsatile pressure from the heart. The elastic properties of the distributing vessels further contribute to the so-called Windkessel effect, transforming a pulsatile flow at the entry into a constant flow at the level of the capillaries. The aorta, pulmonary trunk, carotids as well as the illiac and subclavian arteries are all examples of elastic arteries. As elastic arteries have such fundamental roles in the vascular physiology, any pathology affecting the function of these vessels by inducing a stiffening of their wall may exert dramatic effects on the supply of vital substances to organs. Atherosclerosis principally impacts on large and medium-sized arteries and is the leading cause of mortality worldwide [22]. In brief, the pathogenesis of the disease initiates with the dysfunction of endothelial cells (ECs) characterized by expression of adhesion molecules, secretion of chemokines and increased permeability to low-density lipoproteins (LDL), which will subsequently accumulate in the sub-endothelial space where they get oxidized. The expression of adhesion molecules and secretion of chemokines promotes the entry of inflammatory cells such as monocytes, T lymphocytes and neutrophils into the intimal layer [23,24]. After their infiltration into the intima, monocytes differentiate into macrophages that will take up oxidized LDL and convert it into foam cells. Inflammatory cells secrete metalloproteinases that degrade extracellular matrix (ECM), as well as growth factors stimulating the proliferation and migration of smooth muscle cells (SMCs) from the media to the intima. Intimal SMCs synthesize collagen and their further proliferation eventually leads to the formation of a fibrous cap segregating the necrotic core of the plaque from the luminal blood flow. Plaques with a large necrotic core, a thin fibrous cap, many inflammatory cells and a few SMCs display increased propensity to rupture [25]. Upon rupture, ECM and tissue factor (TF) present in atherosclerotic lesions are exposed to the bloodstream, which initiates a coagulation cascade leading to the formation of a fibrin monolayer covering the site of injury [26,27]. In parallel, the activation of platelet receptors by atherosclerotic plaque components leads to platelet activation and aggregation [26]. If platelet aggregation is not limited, thrombus formation may compromise the arterial lumen and provoke acute ischemic events such as myocardial infarction and stroke.

### 3.1. Connexins and Atherosclerotic Disease

The integrity of the endothelial barrier is warranted by various types of endothelial junctions including gap junctions [28]. During atherosclerotic plaque development pro-inflammatory molecules induce a progressive deterioration of EC junctions and an increase in endothelial permeability. ECs are, for instance, responsive to TNF-α, which induces the expression of adhesion molecules and inflammatory cell recruitment. It has been shown that treating ECs with TNF-α dampens the expression of some Cxs, in particular Cx37 and Cx40, suggesting a possible implication of Cxs in the pathogenesis of atherosclerosis [29]. Moreover, increased vascular permeability is associated with an elevation of Cx43 expression in ECs [30,31]. The first support for the hypothesis that Cxs may participate in the development of atherosclerotic disease came from studies analyzing atherosclerotic lesions at different disease stages in specimen of human, rabbit or mouse origin. In summary, it has been reported that Cx43 was generally absent in ECs of large arteries, but that its expression was induced in ECs at the shoulder region of advanced atherosclerotic plaques, a localization known to experience disturbed blood flow [32]. In addition, high expression of Cx43 was found in macrophages and SMCs of young atherosclerotic lesions, whereas Cx43 levels were downregulated in SMCs of more mature plaques [32,33,34,35]. Interestingly, the oxidized phospholipid derivative 1-palmitoyl-2-(5′-oxo-valeroyl)-*sn*-glycero-3-phosphocholine (POVPC) has been shown to decrease Cx43 expression in SMCs, to increase its phosphorylation, and to promote SMC proliferation in vitro and in vivo in a mouse model of atherosclerosis [36]. Besides Cx43, the expression patterns of Cx37 and Cx40 have also been reported to be affected during atherosclerotic plaque development in humans and in mice [32]. In fact, Cx40 and Cx37 expression was abolished in ECs covering advanced plaques, while Cx37 levels were increased in foam cells. Moreover, long-term hypercholesterolemia in mice decreased Cx37 and Cx40 expression in aortic ECs. Interestingly, this outcome could be reversed exclusively for Cx37 by a one-week treatment with simvastatin, a well-known lipid-lowering drug [37]. Collectively, these observations support the idea that Cxs expression or their post-translational modifications might evolve in atherosclerotic plaques over time, depending on the stage of the lesion, and might thus affect atherogenesis.

As the ubiquitous deletion of Cx43 is lethal [38], *Cx43^+/−^* mice were crossed with atherosclerosis-prone LDL receptor-deficient (*Ldlr^−^*^/*−*^) mice and fed a high cholesterol diet to study atheroma formation. These initial studies revealed that Cx43 has an overall atherogenic effect, and that reducing Cx43 might be beneficial by both reducing plaque burden as well as stabilizing the lesions [39]. However, the exact scenario by which global reduction in Cx43 ultimately led to this dual benefit was unclear, due to Cx43 expression in multiple atheroma-associated cell types. To examine specifically the role of Cx43 in immune cells, *Ldlr^−/−^* mice were lethally irradiated and reconstituted with *Cx43^+/+^*, *Cx43^+/^^−^* or *Cx43^−^^/−^* hematopoietic fetal liver cells [40]. Intriguingly, the progression of atherosclerosis was lower in *Cx43^+/−^* chimeras compared with *Cx43^+/+^* and *Cx43^−^^/−^* chimeras, and their plaques contained fewer neutrophils. It turned out that chemoattraction of neutrophils, which did not themselves express Cx43, was reduced in response to supernatant secreted by *Cx43^+/^^−^* macrophages in comparison with the ones of *Cx43^+/+^* and *Cx43^−^^/−^* macrophages. Thus, titration of Cx43 levels in macrophages might regulate their chemoattractant secretion, leading to reduced atherosclerosis [40]. Recently, it was shown that an upregulation of Cx43 expression in human umbilical vein ECs resulted in enhanced adhesion of monocytes via a mechanism involving increased vascular adhesion molecule-1 and intercellular cell adhesion-1. This effect was independent from the expression of other Cxs such as Cx37 and Cx40 [41].

In contrast to Cx43, Cx40 expression has been reported to protect against atherosclerosis in mice by synchronizing endothelial anti-inflammatory signaling thus inhibiting leukocyte recruitment to the atherosclerotic lesion [42]. Interestingly, Cx40 expression is induced in arterial ECs by high laminar shear stress, as normally observed in straight parts of arteries that are known to be protected from atherosclerosis [43]. IκBα, a member of a protein complex inhibiting the activation of the transcription factor NFκB, was recently identified as a binding partner of Cx40-CT. The Cx40 interactome may be relevant for the control of NFκB activation in arterial ECs and the initiation of atherogenesis [43].

Deletion of Cx37 has been shown to promote atheroma formation in atherosclerosis-susceptible apolipoprotein E-deficient (*Apoe^−/−^*) mice. Mechanistically it was demonstrated that Cx37 hemichannels in monocytes modulate the initial steps of atherosclerosis by regulating their adhesion to the endothelium [44]. Even in later stages of the disease, Cx37 deletion also reduced the stability of shear stress-induced atherosclerotic plaques in *Apoe^−/−^* mice by increasing macrophage contents of the advanced plaques [45]. As the Cx37-CT directly binds to the NO reductase domain of endothelial nitric oxide synthase (eNOS), thereby influencing the function of the enzyme and NO production [46], absence of Cx37 in ECs covering the atherosclerotic lesion may contribute to the dysfunctionality of these cells. Of note, a single nucleotide polymorphism (SNP) in the human Cx37 gene (*Cx37 1019C > T*) associates with an increased risk for coronary artery disease, myocardial infarction, stroke and peripheral artery disease [47]. This *Cx37* *1019C > T* SNP results in a non-conservative Proline-to-Serine substitution in the CT of Cx37 and appeared to have a significant impact on channel function under basal and phosphorylating conditions [46,48,49]. When transfected in HeLa or N2A cells, both polymorphic channels are efficiently transported to the cell membrane, where they may function both as hemi-channels and gap junction channels; however, the unitary conductance of channels formed by the Cx37-Proline isoform appeared 1.5 times larger than the one of the Cx37-Serine isoform [48]. In addition, it was shown that monocytic cells expressing Cx37-319P were markedly less adhesive than cells expressing Cx37-319S. Thus, Cx37-319P polymorphic hemi-channels may function as a protective genetic variant by specifically retarding recruitment of monocytes to human atherosclerotic lesions [44].

Altogether, these studies revealed important and diverse contribution of vascular Cxs to the development of atherosclerosis. Before we may consider Cx-based strategies to fight atherosclerotic disease, more work is needed to discriminate between beneficial effects of reduction of (hemi-) channel function and alteration of the Cx interactome of atherogenesis. Moreover, it remains to be determined whether Cxs may play a role in the mechanisms linked to plaque regression.

### 3.2. Panx1 and Atherosclerosis

**Figure 1:**
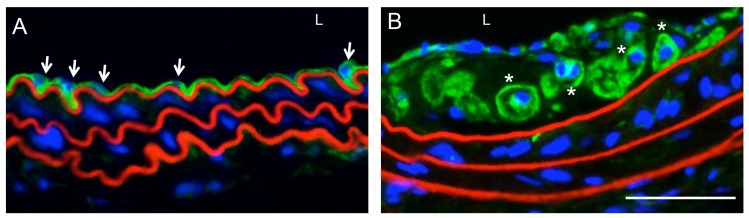
Panx1 expression in healthy and atherosclerotic arteries. (**A**) Panx1 (in green) is expressed in ECs (arrowheads) separating the arterial wall from the lumen (L) of a healthy mouse carotid artery; (**B**) Panx1 is found in lipid-laden macrophages (asterisks) present in atherosclerotic lesions. Of note, Panx1 is absent from the SMC-rich media of non-diseased and diseased conduit arteries. Nuclei are stained with DAPI (in blue) and elastic laminae are counterstained with Evans Blue (in red). Scale bar represents 25 μm.

As illustrated in the next section, Panx1 channels are important regulators of microvascular physiology, mostly through their capacity to release purines, including ATP [50,51]. As such, Panx1 channels were long time hypothesized to play a role in atherosclerotic disease via their effects on inflammasome activation, neutrophil and macrophage chemotaxis and the activation of T cells [52]. Moreover, Panx1 may play a potential role in macrophage apoptosis and clearance from atherosclerotic lesions by allowing the release of “find me” signals from apoptotic cells to recruit phagocytes at the initial steps of programmed cell death [53,54,55]. Examination of Panx1 expression in carotid arteries of *Apoe^−/−^* mice fed with high cholesterol diet revealed Panx1 in the arterial endothelium and in macrophage foam cells in atherosclerotic lesions, and confirmed its absence in the SMCs of the media in these large arteries [56] (Figure 1).

To investigate the potential contribution of Panx1 in endothelial and monocytic cells to atherosclerosis, mice with a conditional deletion of Panx1 were generated. Atherosclerotic lesion development in response to high cholesterol diet was enhanced in *Tie2-Cre^Tg^Panx1^fl/fl^Apoe^−/−^* mice as compared to *Panx1^fl/fl^Apoe^−/−^* controls, pointing to a protective role for Panx1 in endothelial and/or monocytic cells in atherosclerosis. Unexpectedly, atherogenesis was not altered in mice with ubiquitous Panx1 deletion (*Panx1^−/−^Apoe^−/−^*), but these mice displayed reduced body weight, serum cholesterol, triglycerides (TG) and free fatty acids (FFA), suggesting altered lipid metabolism in mice with ubiquitous Panx1 deletion. As it is well known that lowering serum cholesterol and TG levels protects against atherosclerosis in human, it was hypothesized that the lack of effect of ubiquitous deletion of Panx1 on the extent of atherosclerosis may be explained by simultaneous opposite effects of Panx1 on lipid metabolism and inflammation. Interestingly, Panx1-deficient mice show impaired lymphatic function [56] (see Section 6). Future work should unravel the mechanisms linking the lymphatic system, lipid metabolism and atherosclerosis.

## 4. Coordination of Microvascular Function by Gap Junctions

The arterial vascular system supplies oxygen and nutrients to peripheral tissues by controlling blood flow distribution through a complex network of vessels. Resistance to blood flow is a function of the lumen diameter of the vessels, which depends on the degree of vascular smooth muscle constriction (i.e., vasomotor tone). Most of the total resistance to blood flow resides on feed arteries and arterioles; therefore, coordination of changes in vasomotor tone in the microvascular network plays a central role in the regulation of blood flow distribution and arterial blood pressure [57].

The endothelium plays an essential role in the tonic control of vascular function by Ca^2+^-dependent production of vasodilator signals such as NO and prostaglandins [58,59,60]. Although NO is the primary endothelium-dependent vasodilator signal in large conduit vessels, the inhibition of NO or prostaglandin production only attenuates the relaxation initiated by endothelium-dependent vasodilators in small resistance arteries [61,62,63]. The NO- and prostaglandin-independent response observed in these arteries is associated with the hyperpolarization of SMCs, which leads to smooth muscle relaxation by the consequent reduction in the open probability of L-type voltage-dependent Ca^2+^ channels. In addition to the complex EC signaling, the appropriate control of blood flow distribution also relies on the direct cell-to-cell communication via gap junctions, which has emerged as a key pathway to coordinate vascular wall function in resistance arteries by radial (among ECs and SMCs) and longitudinal (along the vessel length) conduction of vasomotor signals [62,64,65,66].

### 4.1. Radial Conduction in the Vascular Wall

Gap junctions play a central role in the intercellular communication of the endothelium-generated vasodilator signals. Although ECs and SMCs are physically separated by the internal elastic lamina in resistance arteries, these cells can make contact through cell projections that penetrate the internal elastic lamina and reach the other cell type at discrete points known as myoendothelial junctions [62,67,68,69]. These points of contact appear to constitute highly specialized subcellular signaling microdomains and gap junctions located at myoendothelial junctions (i.e., myoendothelial gap junctions) provide a critical pathway for fine regulation of vasomotor responses through the radial transmission of current, Ca^2+^ and small signaling molecules such as IP_3_ [68,70,71,72].

The endothelium-mediated NO-independent smooth muscle hyperpolarization was first attributed to a diffusible factor released by ECs and, in consequence, this vasodilator signal was termed endothelium-derived hyperpolarizing factor (EDHF) [61,62]. Several EDHF candidates have been proposed, such as K^+^ ions [73], epoxyeicosatrienoic acids [74,75], hydrogen peroxide [76], and C-type natriuretic peptide [77,78]. Although the NO-independent smooth muscle hyperpolarization is likely to rely on a combination of these signals, depending on the vascular territory [61,79] and experimental preparation used in the study [80], this vasodilator component is however typically paralleled by the hyperpolarization of ECs [61,62]. In addition, it has been consistently observed that the endothelium-dependent smooth muscle hyperpolarization is sensitive to simultaneous inhibition of Ca^2+^-activated K^+^ channels (K_Ca_) of small (SK_Ca_) and intermediate (IK_Ca_) conductance [61,63,81]. In the vessel wall, these K^+^ channels are only expressed in ECs [81,82], which prompted the proposal that a prominent component of the EDHF signaling is the simple direct electrotonic transmission from ECs to SMCs via myoendothelial gap junctions of a hyperpolarizing current initiated by SK_Ca_ and IK_Ca_ activation [61,81,83,84,85]. In which case, the release of a diffusible factor is not consistent with this signaling mechanism, which led to replacing the term abbreviated as EDHF with the expression endothelium-derived hyperpolarization (EDH) [86]. Consistent with this notion, the contribution of the EDH-mediated responses and the expression of myoendothelial gap junctions increase as the vessel size decreases [87,88] and the EDH-associated vasodilator signaling has been shown to be attenuated or abolished by the Cx-mimetic peptides ^37,40^Gap26, ^40^Gap27 and ^37,43^Gap27 [89,90]. These peptides are homologous to specific domains of EL1 (Gap26) or EL2 (Gap27) and were designed to block channels formed by Cx37 or Cx40 in the case of ^37,40^Gap26, Cx40 in the case of ^40^Gap27, and Cx37 or Cx43 in the case of ^37,43^Gap27. In addition to these findings, EC-selective loading with antibodies directed against the carboxyl-terminal region of Cx40 [91] or deletion of Cx40 specifically in ECs also leads to a reduction in the EDH pathway [80], which highlight the functional relevance of this Cx in the endothelial cell signaling and in the control of vasomotor tone.

Interestingly, a pool of eNOS is also found at myoendothelial junctions [92], which provide a subcellular location that is coherent, not only with the vasodilator function of the enzyme, but also with the intercellular signaling pathway of NO. Although the biophysical properties of NO are compatible with the assumption that it can diffuse freely across cell membranes, blockade of gap junction communication in mesenteric resistance vessels with 18β-glycyrrhetinic acid was shown to prevent the NO transfer from ECs to SMCs and the associated NO-dependent vasodilation observed in response to acetylcholine (ACh) [93], suggesting that myoendothelial gap junctions provide a directional pathway for effective NO signaling in the wall of small arteries. The Cx isoforms involved in the gap junction-mediated NO signaling have not been identified, but, as NO-induced relaxation is mediated by a reduction in the Ca^2+^ sensitivity of smooth muscle contractile machinery [94,95] and EDH signaling decreases the intracellular Ca^2+^ concentration of SMCs [61,94], regulation of myoendothelial gap junctions may play a pivotal role in the balance of these two complementary vasodilator components.

### 4.2. Longitudinal Conduction of Vasomotor Responses

Control of peripheral vascular resistance and blood flow distribution is a dynamic process that depends on coordination of changes in diameter between different segments and cellular elements of the vascular resistance network [57,96]. Vasomotor signals generated in a short arteriolar segment (100 µm) rapidly spread (<1 s) several millimeters along the vessel length without apparent delay, demonstrating functional coupling between distal and proximal segments of the vasculature [97,98]. Therefore, longitudinal conduction of vasomotor signals endows the microvascular network with a mechanism that is most likely to contribute to integrate function within the arteriolar network and between arterioles and feed arteries [96,99,100]. Direct measurements of membrane potential indicate that conducted vasomotor responses are associated with changes in the membrane potential of cells of the vessel wall [97,101,102]. As gap junctions provide a low-resistance intercellular pathway between ECs and SMCs, the conduction of vasomotor responses along the vessel length is thought to be the result of electrotonic spread of changes in membrane potential generated at the stimulation site through gap junctions connecting cells of the vessel wall [103,104]. Then, in the case of endothelium-dependent vasodilators, such as ACh, the conduction of the vasodilation is thought to be the result of the electrotonic spread along the vessel length of an EDH-initiated vasodilation [62,64,105]. In contrast, in the case of vasoconstrictor signals, such as those activated by phenylephrine (PE), a depolarization is conducted [101,106].

The cellular pathway of conducted vasomotor signals seems to depend on the cell type that initiates the response, and vasoconstrictor responses activated by the stimulation of SMCs are consistently conducted by SMCs [107,108]. In contrast, vasodilator signals have been shown to spread either exclusively by the endothelium in feed arteries [97,109] or by both SMCs and ECs in arterioles [107,108], which led to the proposal that the cellular pathway for conduction of vasodilations depends on the functional location of the vessel in the microvascular network [66]. However, the cellular pathway of vasodilator signals may also depend on the stimulus that initiated the response, because, in contrast to ACh, selective damage of the endothelium blocked the vasodilation induced by bradykinin in arterioles [104,108].

Conduction of vasomotor responses may be mediated by interaction of one or more of the five Cx isoforms that are expressed in the vascular system: Cx32, Cx37, Cx40, Cx43, and Cx45 [100,110,111,112]. Although the contribution of each of these Cxs to the longitudinal coordination of the changes in diameter has not been clearly determined, it has been consistently observed that global deletion of Cx40 results in the development of an irregular arteriolar vasomotion and in a reduced spread of vasodilator signals activated by ACh or bradykinin in feed arteries as well as in arterioles of the cremaster muscle microcirculation [98,113,114]. In blood vessels of the mouse, the expression of Cx40 is restricted to the endothelium [98,115,116], which raises an apparent disagreement with the participation of SMCs in the conducted vasodilation in arterioles. However, the involvement of Cx40 in the transmission of the EDH signaling may explain the detriment of the alternative conduction through SMCs observed previously in response to ACh [80]. In addition, ablation of Cx40 is also associated with a decrease in Cx37 expression and the development of a hypertension caused by a dysregulation of renin production [98,99,114,116,117]. As with Cx40, the expression of Cx37 is also confined to ECs in the vessel wall of mice [98,116], and then, the decline in Cx37-mediated communication in the absence of Cx40 and the development of hypertension may contribute to the reduction in the conduction of vasodilator signals observed in Cx40 knockout animals. Nevertheless, conducted vasodilator responses are intact in Cx37 knockout mice [98] and in animals with an angiotensin-dependent hypertension evoked by deletion of Cx40 in the renin-producing cells [116]. Furthermore, the disruption in the propagation of the response to endothelium-mediated vasodilators attained after global deletion of Cx40 was also observed in EC-specific Cx40 knockout mice [116] and in animals expressing a mutated Cx40 (Cx40A96S) that exhibits a substantially lower junctional conductance [116,118,119,120]. Although the mutation Cx40A96S causes a renin-dependent hypertension, as that observed with global deletion of Cx40, the endothelial Cx37 levels are normal in these mice [116]. Therefore, these findings in conjunction confirm the critical role of Cx40 in the control and coordination of microvascular function by ECs.

## 5. Coordination of Microvascular Function by Pannexins

There are three different pannexin isoforms (Panx1, Panx2 and Panx3), with Panx1 being the most ubiquitously expressed throughout the vasculature [121]. There are organ-specific circulations where it appears that other Panx isoforms have been described, but their function has not yet been described [121]. In general, Panx1 is expressed in endothelium throughout conduit and microcirculation, whereas Panx1 is restricted to smooth muscle of resistance arteries, and is not found in conduit smooth muscle [56,121]. Overall, much less is known about the Panxs (compared with Cxs) in the microcirculation, likely due to their more recent discovery, the inherent problems associated with the global Panx1 knockout mouse (e.g., compensation with up regulation of Panx3 throughout the vasculature [122], as well as other cell types [123]), and specific inhibitors for Panx1 that do not also block connexin-built gap junctions (e.g., [124]). However, there are exciting pieces of data emerging using inducible cell type specific Panx1 knockout mice that have revealed phenotypes that are fundamental to the microcirculation.

For example, multiple groups have now demonstrated that Panx1 and the α1-adrenergic receptor (AR) are uniquely coupled in a signaling axis that can regulate vasoconstriction [125,126,127,128,129]. Either SMC-specific Panx1 deletion, or use of multiple Panx inhibitors, blunts noradrenaline and PE mediated vasoconstriction of resistance arteries, but leaves other vasoconstriction pathways intact [125,127,128,129]. This translates to a hypotensive blood pressure response by the mouse at periods of highest sympathetic nerve activity (evening) [130]. Importantly, the Panx1-α1-AR signaling axis is not observed in large conduit arteries (e.g., aorta or carotid), which is likely because Panx1 is absent from conduit vessel smooth muscle [56,121], and sympathetic nerve innervation is very low.

The Panx1-α1-AR signaling axis also highlights a potent link between sympathetic nerves and vasoconstriction that may be directly druggable for treatment of hypertension in humans (e.g., [127,129]). Indeed, this was recently highlighted by the discovery of trovafloxacin and spironolactone being able to work directly on Panx1 channels [127,131], as evidenced by electrophysiology, inhibiting ATP release, and blunting of vasoconstriction. Spironolactone in particular has been used for decades as a potent anti-hypertensive whose primary effect had been thought to be due to mineralcorticoid antagonism. Other more specific mineralcorticoid antagonists failed to block the Panx1 channel, indicating that the potent effect of spironolactone may be due to blocking both mineralcorticoids and Panx1.

The mechanism of α1-AR activation of Panx1 is still under investigation, although based on previous work it is thought that Panx1 may be selectively regulated by receptor stimulation at the intracellular loop [130]. The use of both peptides and amino acid mutagenesis have confirmed the importance of this region [130]. However, there are likely other regions where Panx1 can be regulated that are especially important in the vasculature. For example, NO potently inhibits Panx1 channels by S-nitrosylating amino acids cysteine 40 and cysteine 346 to prevent channel opening and ATP release [132]. This could be an important mechanism for feedback on sympathetic nerve vasoconstriction. How the cross-talk of several different post-translational modifications fit together to regulate Panx1 channel gating properties will be important moving forward.

There are other more specific regions where Panx1 may play a role in the microcirculation. There is no identified role yet for Panx1 in regulation of endothelial-mediated dilation, except in large conduit vessels, which do have augmented responses to endothelial-induced vasodilation in global Panx1 knockout animals [133]. However, this effect is not seen in resistance arteries, and endothelial specific deletion of Panx1 has no effect on blood pressure [134]. Thus, it is not clear what exactly the augmented endothelial mediated dilation in conduit arteries may mean physiologically at this point.

Panx1 utilization could also be considered vascular bed specific. For example, it has recently been demonstrated that myogenic tone is attenuated in the cerebral circulation of EC-specific Panx1 knockout animals, but is not altered in the mesenteric circulation of the same animals [134]. The EC- specific Panx1 knockout mice also had resistance to middle cerebral artery occlusion (stroke model). SMC-specific Panx1 knockout animals did not have an attenuation of myogenic tone in the cerebral or mesenteric circulation, and were not resistant to induction of stroke [134]. Thus, different vascular beds may utilize Panx1 differently. It also highlights the importance of using cell type-specific Panx1 knockouts in order to properly identify phenotypes.

Besides regulation of blood pressure, among other important aspects of the microcirculation, it is an important regulator of the acute inflammatory response. It was recently demonstrated that TNFα stimulation activates Panx1 in the venous, but not the arterial microcirculation [135]. Interestingly, TNFα (but not IL-1β) induced ATP release via Panx1, as demonstrated in cultured venous ECs, as well as isolated murine veins, but not in any arterial EC or isolated arteries [135]. The effect of the increased ATP release caused an increase in leukocytes that was Panx1 dependent as shown by genetic deletion [135]. The effect of EC deletion of Panx1 has also recently been shown in ischemia, with deletion of Panx1 inducing a significant decrease in leukocytes after occlusion of the middle cerebral artery, lessening the overall impact of the ischemic response [136]. Recently, this work was even further expanded to include ischemic models in the lung and kidney [137,138]. These exciting observations point to a central role for Panx1 in ECs regulating ischemia and the acute inflammatory response.

Also, in the microcirculation, there has been significant attention paid to the possible role of purinergic signaling from red blood cells (RBCs) to endothelium to induce vasodilation, especially during hypoxia. It had been hypothesized that Panx1 on RBCs was the mechanism by which ATP (or other purinergic signals) could leave the RBC, bind to purinergic receptors on endothelium, and induce vasodilation. However, although Panx1 can be localized to RBCs [136], the role for ATP coming from RBCs has recently been called into question, especially via activation of the channel by cAMP/PKA [136,139]. Indeed, the mechanism for increased ATP may be lysis of the RBCs [136,139]. This highlights the need to be careful with measurement of ATP, which is an inconsistent and difficult methodological technique. However, further questions that arise based on this potential heterocellular communication between RBCs and endothelium include what the possible role of Panx1 on RBCs would be if it was present, or if other signaling mechanisms besides cAMP could induce Panx1 channel opening on RBCs.

## 6. Role of Connexins and Pannexins in Venous and Lymphatic Function

Apart from their role in inflammatory cell recruitment at the level of venules (see Section 5), the function of Panxs and Cxs in larger veins is much less studied. Venous valves play a crucial role in the systemic circulation, promoting the one-way movement of blood from peripheral veins towards the heart and augmenting venous return. In humans, valvular dysfunction or (congenital) absence of valves in large veins typically result in common venous disorders such a varicose veins and edema in the legs. Three gap junction proteins, i.e., Cx37, Cx43, and Cx47, are expressed in ECs, covering venous valves in a highly polarized fashion, with Cx43 on the upstream side of the valve leaflet and Cx37 on the downstream side. Cx47 seems more restricted to a small subset of ECs in the venous valves [140]. Similar to earlier observations in the lymphatic vasculature [141,142] veins from Cx37-deficient mice lack valves [140]. As Cx37 seems a crucial regulator of valve development in both veins and lymphatic vessels, there may be common molecular pathways controlling valve development in these distinct vessel types. Mechanistically, it has been shown in lymphatic valves that the transcription factors Prox1, Foxc2, as well as lymphatic flow, coordinately control the expression of Cx37 and activation of calcineurin/NFAT signaling. Indeed, Cx37 and calcineurin are required for the assembly and delimitation of lymphatic valve territory during development and for its postnatal maintenance [142]. Interestingly, the development of venous valves, but not the formation of lymphatic valves, is affected in Cx47-deficient mice [143,144]. Accordingly, Cx47 null mice also display normal lymphatic vascular function [145]. Mutations in the Cx47 gene are associated with reduced venous valve number and length, a crucial finding for understanding how some Cx47 mutations cause inherited (lymph) edema in humans [144]. Recently, both Cx47 and Cx43 have been added to the limited repertoire of primary lymphedema-associated genes (such as Foxc2, Vegfr3 and Sox18) [146,147,148,149]. Furthermore, Cx47 and Cx37 mutations have been associated with increased risk for secondary lymphedema following breast cancer treatment [150,151].

The lymphatic system regulates tissue fluid homeostasis, trafficking of immune cells to draining lymph nodes and absorption of dietary fat. To investigate whether Panx1 affects lymphatic flow, drainage of interstitial fluids following injection of Evans Blue in the footpad was recently compared between *Panx1^−/−^Apoe^−/−^* and *Apoe^−/−^* mice. The dye progressively spread throughout the lymphatic system to successive draining lymph nodes and finally the systemic circulation. The dye transport was considerably smaller in *Panx1^−/−^Apoe^−/−^* mice than in control *Apoe^−/−^* mice. Moreover, tails of *Panx1^−/−^Apoe^−/−^* mice showed increased diameters and increased interstitial fluid content than control *Apoe^−/−^* mice, suggesting that lymphatic flow is impaired in mice with ubiquitous deletion of Panx1. Finally, *Panx1^−/−^Apoe^−/−^* mice had reduced dietary fat absorption with control animals. Collectively, these findings suggest a pivotal role for Panx1 in lymphatic function [56], and it will be exciting to learn more on the cell type and molecular mechanism involved in this regulation.

## 7. Connexins and Pannexins in the Control of Platelet Function, Haemostasis and Thrombosis 

Cx hemichannels and gap junctions have been studied widely in various cell types where sustained cell interactions occur. Some reports, however, indicate the presence of Cxs on the surface of some circulating cells, such as monocytes and T-cells, where gap junction and hemichannel functions control cellular functions [152,153,154]. In recent studies, fundamental roles for these proteins in platelets have emerged.

### 7.1. Platelets: Mediators of Haemostasis and Thrombosis

**Figure 2:**
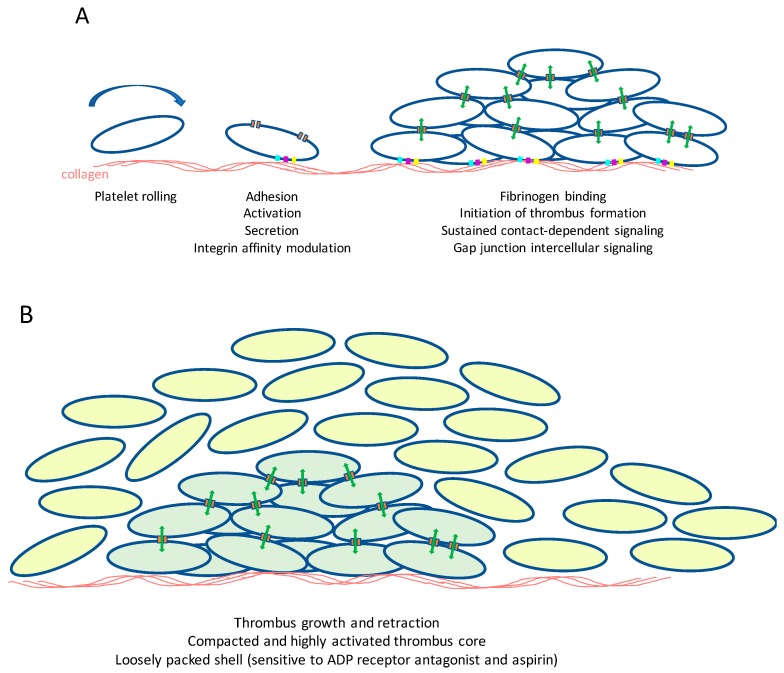
Gap junction intercellular communication between platelets: a working model. (**A**) Blood vessel injury leads to the exposure of subendothelial collagens. Through interaction with von Willebrand factor, which also binds to collagens, platelets roll along the surface, slowing their movement and allowing direct binding of collagen to the cell surface receptors integrin, α_2_β_1_, and GPVI, initiating platelet intracellular signaling. This results in the secretion or release of prothrombotic factors such as ADP and TXA_2_ that further propagate platelet activation. This culminates in an increase in affinity of integrin α_IIb_β_3_ which then binds fibrinogen that supports platelet-platelet adhesion and thrombus formation. Close contact between platelets allows the formation of gap junctions that permit intercellular signaling during thrombus formation and stabilization; (**B**) Intercellular signaling controls thrombus contraction by enabling the formation of a core of platelets that are highlight activated and tightly packed. A more loosely packed shell of platelets develops, although this is inhibited in the presence of ADP receptor antagonists or aspirin (to prevent TXA_2_ formation). Whether gap junctional intercellular communication controls platelet thrombus core and shell assembly has yet to be formally established.

Platelets provide a front line of defense in response to injury, triggering haemostasis at sites of injury, and are increasingly recognized for their involvement in a range of other (patho)physiological processes, including inflammation, atherogenesis, and cancer cell metastasis. Platelets adhere to collagens that are exposed at sites of arterial damage, initially via an indirect interaction with plasma von Willebrand factor (VWF), which through binding to the platelet glycoprotein (GP) Ib-V-IX receptor complex, a short-lived interaction that therefore serves to slow platelets, allows subsequent direct binding to platelet collagen receptors GPVI and integrin α_2_β_1_ (Figure 2A) [155,156]. Integrin α_2_β_1_ functions principally as an adhesive receptor for collagen [157], while collagen binding to GPVI stimulates platelet activation.

Collagen binding to GPVI causes receptor clustering and the tyrosine phosphorylation of the Fc receptor γ-chain [158,159] by Src-family kinases [160]. The tyrosine kinase Syk is then recruited and initiates the first step in a complex and branching signaling pathways incorporating, among others, the linker for activation of T cells, phosphatidylinositol 3-kinase, protein kinase B, Bruton’s tyrosine kinase, phospholipase Cγ2, integrin-linked kinase, and the mobilization of intracellular calcium stores [161,162]. This culminates, via the GTP binding protein Rap1b, in an increase in affinity of integrins that enhance adhesion to collagen (α_2_β_1_), and causes aggregation through the binding of plasma fibrinogen to integrin α_IIb_β_3_ [161,163].

A rapid and full platelet response is ensured through the autocrine and paracrine actions of factors that are released by activated platelets such as ADP and thromboxane A_2_. Following the binding of fibrinogen to integrin α_IIb_β_3_ and collagen to integrin α_2_β_1_, outside-in signaling through these integrins also contributes to sustained platelet activation and irreversible thrombus formation [164,165]. Thrombin, which is generated on the surface of activated platelets within a thrombus, is also a potent platelet agonist that is important for effective haemostasis.

A reactive system such as this, which incorporates many positive feedback mechanisms, requires precise control to prevent un-needed platelet activation. The healthy endothelium produces molecular signals, NO and prostaglandin I_2_, short-lived molecules that exert powerful inhibitory effects on platelets through the stimulation of cyclic nucleotide-dependent intracellular signaling [166].

Inappropriate platelet activation, for example, at the site of atherosclerotic plaque formation or rupture, results in the exposure of platelets to activatory substances, including collagens, resulting in thrombosis and the occlusion of blood flow. As the principle cause of myocardial infarction and ischemic stroke, platelets represent an important therapeutic target [163].

In the last 20 years, substantial progress has been made in understanding the molecular mechanisms that control platelets, and this is beginning to impact in the development of new therapeutic approaches. These advances have largely involved study of traditional intra-cellular signaling, the identification of key activatory or inhibitory signals, cell surface receptors required to respond to these signals and the intracellular signaling pathways or networks that these control. While platelets are singular circulating cells, activation and thrombus formation bring them into close proximity for prolonged periods, and increasing evidence supports the importance of sustained inter-platelet communications within the thrombus derived largely through integrin outside-in signaling, with additional contributions from, for example, Eph family kinases and ephrin counter-ligands [167,168,169]. Sustained signaling within the thrombus enables platelets to cooperate to regulate thrombus compaction, structure and stability and subsequently clot retraction, a step believed to be important for wound repair [170]. The ability of platelets to coordinate their functions within a developing thrombus, particularly in the control of calcium signaling, led to early experimental evidence that this may be mediated by intercellular communication (Figure 2B), although this was not initially attributed to gap junctions [171].

### 7.2. Platelets Possess Connexins

Messenger RNA profiling of megakaryocytes, the precursor bone marrow cells from which platelets form, revealed that these cells likely possess notable levels of Cx37, with additional expression of Cx40 and Cx62. Indeed, Cx37 mRNA and protein were first reported to be expressed in human and mouse platelets, and were found to be present at the cell surface [172,173]. Scanning electron microscopy of sections of human platelet thrombi revealed regions of apposite membrane structures with a typical appearance of gap-junction-like structures [173].

### 7.3. Platelet Gap Junction Formation—Orchestration of Intercellular Signaling within Arterial Thrombi

The transport of dye between platelets was first demonstrated following micro-injection of neurobiotin, and transfer into surrounding platelets [172]. Gap junctional intercellular coupling between platelets was confirmed by fluorescence recovery after photo-bleaching (FRAP) analysis of thrombi preformed under arterial flow conditions using blood reconstituted with platelet that were labelled preloaded with cytosolic calcein [173]. Transfer of dye was inhibited by selective or non-selective inhibition of Cx37 (^37,43^Gap27). Clot retraction responses were also inhibited in the presence of inhibitor, or the absence of Cx37 (using blood from Cx37-deficient mice) suggesting that gap junctional coupling mediates physiological responses within platelets.

There currently exists a difference of experimental conclusions drawn from the study of Angelillo-Scherrer, who reported modestly elevated platelet aggregation responses on Cx37-deficient mouse platelets and following the use of inhibitory peptides with human platelets [172], while Vaiyapuri reported substantially diminished responses [173]. Vaiyapuri also reported similar outcomes following the inhibition or deletion of Cx40 [173]. These differences are likely to be explained by differences in experimental conditions, although both studies indicate the potential importance of Cxs in the control of platelet function.

The use of flow cytometry gated to examine the function of individual platelets revealed that platelet activation, prior to platelet-platelet contact is inhibited in the absence of functional Cx37, which is suggestive of important roles for Cx hemichannels in the initiation of platelet responses [173,174]. Whether this is due to channel function or through interaction with other cell surface proteins has yet to be established, although inhibition of Cx37 or Cx40 is associated with diminished intracellular mobilization of calcium from stores, indicating a fundamental role in the propagation of platelet cell signaling [173,174]. Consistent with this, inhibition of Cx37 in whole blood results in diminished thrombus formation on a collagen-coated micro-fluidic flow cells under arterial flow conditions. Infusion of ^37,43^Gap27 [173] or ^40^Gap27 (unpublished observation) into mice prior analysis of laser induced thrombosis in cremaster muscle arterioles was found to result in diminished thrombotic responses. Diminished thrombus formation appears to be at the expense of haemostatic control, since bleeding times were extended modestly. It is interesting to note the Cx37-deficient mice were found to exhibit reduced survival time using a thrombo-embolism model, induced by intravenous injection of collagen and adrenalin [172], which may be a consequence of reduced thrombus stability that results in increased thrombus fragmentation and lung occlusion.

### 7.4. Panx1 Contributes to Platelet Function at Low Agonist Concentrations

Recent analysis of the megakaryocyte (and therefore the platelet) channelome revealed that among a range of cell surface channel proteins, Panx1 is also likely to be expressed. A series of pharmacological approaches using selective mimetic peptide inhibitor [175], subsequently confirmed using Panx1-deficient mouse platelets [176], demonstrated this protein to contribute to calcium responses to low concentrations of various platelet agonists. The ability of Panxs to facilitate the release of ATP from cells is a property that has also been observed in platelets. Panx1 mediated ATP release results in subsequent stimulation of the ATP-gated calcium channel P2X1, which causes enhanced calcium influx and therefore propagation of platelet functional responses [175,176]. At higher concentrations of collagen or other platelets agonists P2X1 makes little contribution to cell responses, and therefore the effects of Panx1 are restricted to conditions where agonist concentrations are limited.

### 7.5. Cx37 and Panx1 Polymorphisms

It is fair to ask, are the effects of platelet Cxs likely to be physiologically important, or do they contribute to cardiovascular disease risk? The clearest indication that gap junction and hemichannel function may be important stem from studies that have explored the effects of common gene SNPs in the human population. As discussed before (Section 3), a SNP in the coding sequence of Cx37 (P319S) influences the gating of Cx37 channels [46,48]. When transfected into HeLa cells, the 319P polymorphism is associated with reduced diffusion of dye between cells [172]. 96 Caucasian men were genotyped to explore the relationship between this polymorphism and platelet function. A relationship was observed between the number of 1019C alleles of the *Cx* gene possessed by volunteers, with the CC genotype associated with modestly increased platelet function [172]. These data suggest that platelet reactivity levels may be determined by connexin-mediated platelet function.

Three variants exist in the human Panx1 coding sequence. These result in a Q5H variant at the N-terminus, a I272V variant within the 4th transmembrane domain and deletion of amino acids 401 to 404 at the CT due a splice variant. In a population of 96 male Caucasian volunteers the splice variant was not detected [176]. Two thirds of subjects possessed the allele coding for the histidine variant at position 5 in the protein sequence, and the associated with a small increase in platelet aggregation in response to collagen, although responses to other agonists were unaltered. Comparison between the Panx1 alleles responsible for variability at position 272 showed no relationship with platelet reactivity.

The numbers of subjects included in these studies were relatively small, and more detailed analysis of the relationship between platelet Cx and Panx polymorphisms and variability in platelet responsiveness would be required to confirm these observations. Current data are, however, consistent with a role of Cxs and Panxs in the regulation of haemostasis and potentially thrombosis, allowing the potential development of new strategies for the prevention of thrombosis, or other conditions in which these cells are implicated.

## 8. Conclusions and Perspectives

It is now well established that Cxs have an important function in the control of blood flow distribution and tissue homeostasis, as well as in pathologies that involve a tight regulation and coordination between cells in the blood vessel wall and circulating blood cells such as atherosclerosis and hypertension. Strong evidence now also supports an important role for gap junctions and Cx hemichannels in the control of haemostasis and thrombosis, although many questions remain to be addressed in this field. More insight into the nature of molecular signals that are transported through gap junctions and hemichannels will be required in order to tease apart the basis of their ability to modulate diverse aspects of vascular function. Systems biology approaches have revealed exquisite detail regarding the architecture of platelet thrombi, which are organized into a densely packed core, surrounded by a less densely packed shell, which is sensitive to the actions of anti-platelet drugs [177]. It is possible that gap junctional intercellular communication mediates the organization of thrombus architecture, function and sensitivity to anti-thrombotic medication. The ability of gap junctions to support interactions between different cell types that are implicated in the stimulation of localized inflammatory responses and atherogenesis [44,178,179], further supports the notion that gap junctional coupling between different vascular cell types may impact on a wide(r) range of (patho)physiological processes.

The release of ATP or Ca^2+^ are generally assumed to be the most relevant signaling mechanisms mediated by both Cx hemichannels and Panx1 channels in vascular (patho)physiology. While recent years have shown great progress in the knowledge on Cx43 protein domains involved in gap junction channel vs. hemichannel gating [180], one of the inherit problems with Panxs is that the biophysical properties of the channels are still being worked out, and thus technically there remains a significant number of unknowns. For example, does ATP always come out of a channel? There is no reason that this needs to be the case, as Panxs in general are large pore channels. Also, being able to distinguish between receptor-mediated and caspase cleavage of the channel has become an important technical differential. The physiological effects described in Section 5 would be considered receptor-mediated Panx1 channel opening, which is uniquely different than caspase cleavage-mediated Panx1 opening that occurs during apoptosis. Key physiological parameters such as electrophysiology and ATP release can be observed in both receptor- and caspase cleavage-mediated Panx1 channel opening. The difference being that receptor mediated Panx1-channel opening is transient, and caspase cleavage produces a permanently “open” channel [126]. Perhaps differential dye uptake could help differentiate these two events? Whatever the case, there is still a significant amount to learn about the relatively recently discovered Panx1 channel and how it may affect vascular and platelet function.

## Abbreviations

Cx	Connexin
Panx	Pannexin
TM	Transmembrane
EL	Extracellular loop
CT	Carboxy-terminus
NT	Amino-terminus
IL	Intracellular loop
ER	Endoplasmic reticulum
EC	Endothelial cell
LDL	Low-density lipoprotein
ECM	Extracellular matrix
SMC	Smooth muscle cell
TF	Tissue factor
POPVC	1-palmitoyl-2-(5′-oxo-valeroyl)-*sn*-glycero-3-phosphocholine
eNOS	Endothelial NO synthase
SNP	Single nucleotide polymorphism
TG	Triglycerides
FFA	Free fatty acids
EDHF	Endothelium-derived hyperpolarizing factor
EDH	Endothelium-derived hyperpolarization
ACh	Acetylcholine
PE	Phenylephrine
AR	Adrenergic receptor
RBC	Red blood cells
VWF	von Willebrand factor
GP	Glycoprotein
FRAP	Fluorescence recovery after photo-bleaching
